# Half-millennium evidence suggests that extinction debts of global vertebrates started in the Second Industrial Revolution

**DOI:** 10.1038/s42003-022-04277-w

**Published:** 2022-12-13

**Authors:** Ziyan Liao, Shushi Peng, Youhua Chen

**Affiliations:** 1 China-Croatia “Belt and Road” Joint Laboratory on Biodiversity and Ecosystem Services, Chengdu Institute of Biology, Chinese Academy of Sciences, Chengdu, China; 2 Sino-French Institute for Earth System Science, College of Urban and Environmental Sciences, and Laboratory for Earth Surface Processes, Peking University, Beijing, China

**Keywords:** Biodiversity, Conservation biology

## Abstract

Extinction debt describes the time-lagged process of species extinction, which usually requires dozens to hundreds of years to be paid off. However, due to the lack of long-term habitat data, it is indeterminate how strong the signal of extinction debts is at the global scale and when the debts started. Here, by compiling the geographical distributions of 6120 reptiles, 6047 amphibians, and 4278 mammals and correlating them with annual forest cover data from 1500 to 1992, we show that the beginning of the Second Industrial Revolution (the mid-19^th^ century) was the earliest signal of cumulative extinction debts for global forest-dwelling vertebrate groups. More importantly, the impact of global protected areas on mitigating accumulated vertebrate extinction debt is not as immediate as that of mitigating reduced forest cover but rather suffers from pronounced time-lag effects. As the disequilibrium of vertebrate richness and forested habitat is currently taking place, preventive actions should be taken to promote a well-balanced status among forest restoration, protected areas, and biodiversity conservation to slow the accumulating debts for global forest-dwelling vertebrates.

## Abstract (web summary)

The extinction debt being paid in modern biodiversity crashes began in the Second Industrial Revolution, indicating a long-term anthropogenic impact on forest species richness.

## Introduction

Species extinctions due to habitat destruction have been widely observed globally^1,2^ and are likely to be exacerbated under rapid climate change in the coming decades^3^. Accurately estimating species loss, which is relevant for coping with the climate change crisis, has become a fundamental and important goal in conservation^4^. The diversified responses of biodiversity, typically coupled with time lags and geographic variability, complicate the prediction of species extinction^5^.

Delayed extinction of species, also called extinction debts^1,6^, greatly influences the accurate estimation of species extinction. The evidence of extinction debt can be detected through multiple empirical methods^7^. Three species–area relationship-based approaches, i.e., ‘past habitat’, ‘past communities’, and ‘stable habitats’^8^, are promising for exploring extinction debt at large scales owing to their advantages in quantifying whole communities rather than single species^9^. As the acquisition of both long-term sequential habitat and biodiversity data is difficult, the ‘past habitat’ approach, although it is not the most effective method, typically has been applied to the detection of short-scale spatiotemporal extinction debts for various taxa^9–13^.

The ‘past habitat’ method identifies the signals of extinction debts by comparing correlations between static present-day species richness and dynamic habitat variables (e.g., area size) from the past to the present^14^. Evidence of extinction debts is supported if the past-habitat predictors better explain the present-day species richness than the current habitat predictors^7^. The underlying assumption of this method is, of course, that species have not shifted widely in their distribution ranges for hundreds of years^8^.

The temporal trend of biodiversity change would not be thoroughly investigated when ecological data are analyzed over short- or long-term periods with only several intermediate time points^15–17^. This is particularly true for extinction debts, which occur and are completed over dozens, if not hundreds, of years^1,18^. Therefore, for deriving convincing evidence of extinction debts and illuminating a comprehensive trajectory of biodiversity losses, long-standing yearly habitat-changing archives spanning several centuries become indispensable and important.

Extinction debts caused by historical habitat loss are extremely relevant for future conservation planning^19,20^. Among different types of natural habitats, forested habitats account for only 30% of the global land surface but sustain nearly 80% of the biodiversity^21^. The global biodiversity crisis is highly pertinent to the destruction and fragmentation of forest habitats^22^. To alleviate such a crisis, a large number of protected areas (PAs) have been established globally over the past two centuries^23^. It remains, however, unclear whether the globally established PAs have a positive effect on mitigating the species extinction debts. In summary, we hypothesized that the debts of forest-dwelling vertebrates probably began with the onset of human industrialization and the massive destruction of forests. In this work, by using the outputs of earth system models at a global scale, we provide half-millennium evidence showing that extinction debts for forest-dwelling vertebrate species (mammals, amphibians, and reptiles) have been accumulating since the mid-19^th^ century. Meanwhile, the contribution of PAs to the maintenance of biodiversity-habitat correlation is not as immediately visible as thought.

## Results

### Historical forest dynamics and contemporary species richness of forest-dwelling vertebrates

**Fig. 1:**
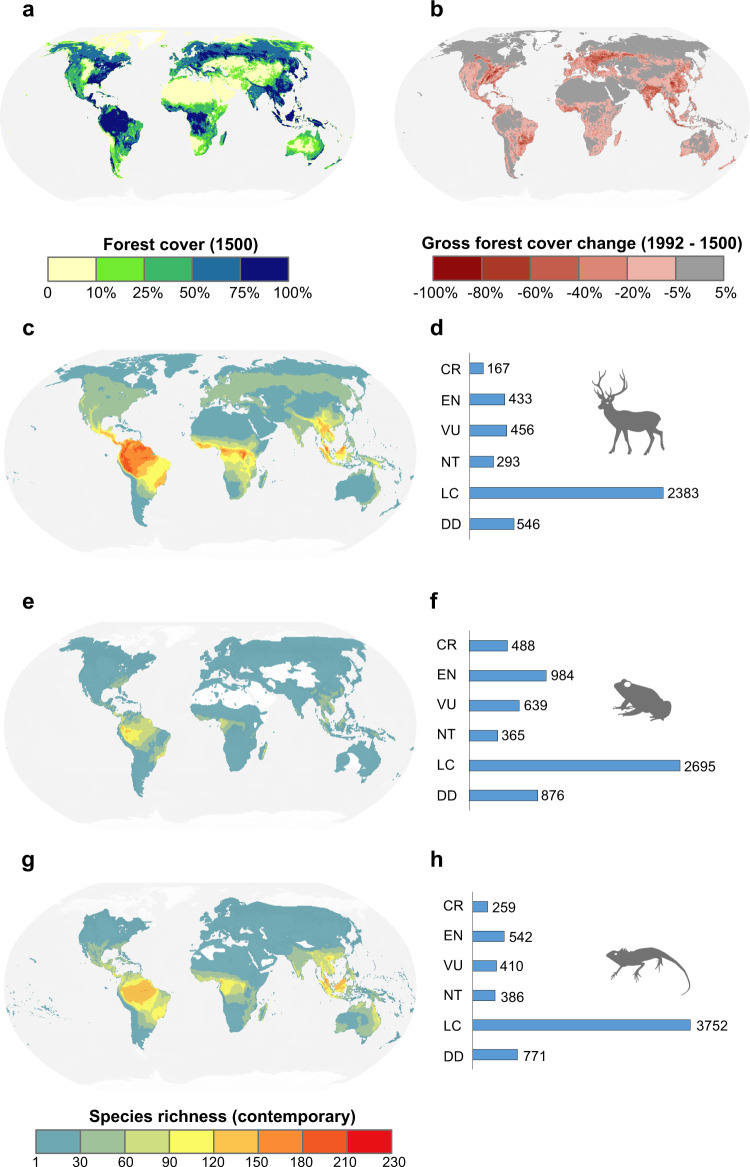
Overview of global forest cover change and forest-dwelling vertebrate species richness. Forest cover for the year 1500 reconstructed using ensemble weighting of backward, forward and JP algorithms **a**. Percent gross forest cover change (unit: %) from 1500 to 1992 **b**. Contemporary species richness of all forest-dwelling mammals **c**, amphibians **e**, and reptiles **g**; The IUCN Red List categories for mammals **d**, amphibians **f**, and reptiles **h** are accompanied on the corresponding right side. *CR* critically endangered, *EN* endangered, *VU* vulnerable, *NT* near threatened, *LC* least concern, *DD* data deficient. The projection system is the Robinson projection, and the base map was obtained from Natural Earth (http://www.naturalearthdata.com/).

Regardless of the reconstruction algorithms used (Supplementary Fig. 1), the vast majority of forested habitats across the globe were consistently found to decline over the past half millennium (Fig. 1a, b). In particular, the historical forest reduction rate was consistently high in many parts of East Asia, Europe, North America, tropical Africa, and southeastern South America, reaching nearly −2.0% per decade (Fig. 1b). Species richness hotspots for forest-dwelling mammals (Fig. 1c), amphibians (Fig. 1e), and reptiles (Fig. 1g) are largely concentrated in tropical and subtropical regions such as South America, Central Africa, and Southeast Asia, which have historically maintained substantial forests (Fig. 1a). Threatened forest-dwelling species are mostly represented by amphibians, followed by reptiles and mammals (Fig. 1d, f, h).

### Global evidence of vertebrate extinction debts

**Fig. 2:**
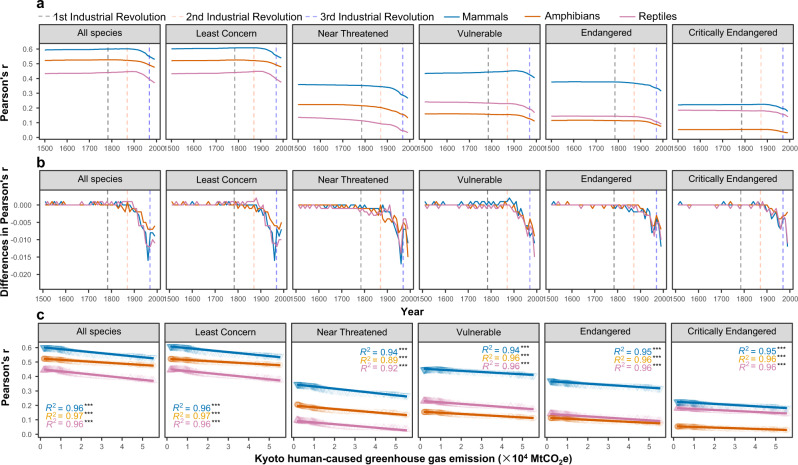
Evidence of vertebrate extinction debts at the global scale. Correlation coefficients (Pearson’s r) per year between the richness of contemporary forest-dwelling vertebrate taxa and the area size of historical forested habitat at the global scale from 1500 to 1992 (*n* = 493) **a**. Difference in correlation coefficients per decade from 1500 to 1992 (*n* = 49) **b**. Linear relationship between yearly correlation coefficients and Kyoto human-caused greenhouse gas emissions (*n* = 143) **c**.

For all three taxonomic groups, before the mid-19^th^ century, at the beginning of the Second Industrial Revolution, the correlations between global forest-dwelling species richness and forest area size were high and stable (Fig. 2a). However, the correlation coefficients decreased gradually from then until the present time (Fig. 2a). Irrespective of the reconstructed forest cover datasets and the categories of IUCN Red List used, the highest correlation was always between mammal richness and forest cover (Fig. 2a and Supplementary Fig. 2). Remarkably, for high extinction risk categories (vulnerable, endangered, and critically endangered), amphibian richness is consistently less correlated with forest cover than is reptile richness; for low extinction risk categories, the opposite is true (Fig. 2a). The change rate of the correlation values between two successive decades further demonstrated the above-described declining trend of the correlation between species richness and the area size of forested habitat (Fig. 2b and Supplementary Fig. 3). There was very strong evidence that the correlation coefficients for the three taxonomic groups were negatively affected by the emissions of human-caused greenhouse gas (all R^2^ ≥ 0.89, *p* ≤ 0.001, *n* = 143) (Fig. 2c).

### Regional evidence of vertebrate extinction debts

**Fig. 3:**
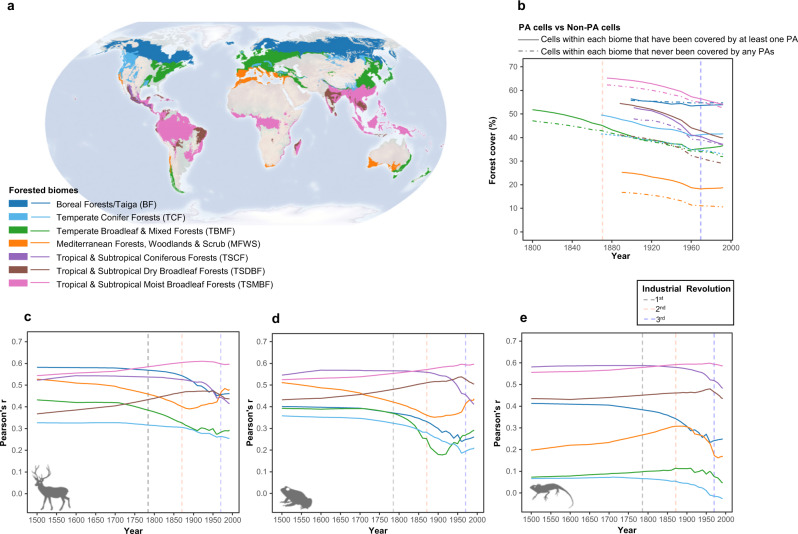
Evidence of vertebrate extinction debts at the regional scale over seven forested biomes. The distributions of the seven forested biomes **a**. Comparison of the forest cover changes between the cells that have been covered by at least one protected area and the cells that have never been covered by any PAs **b**. Annual change in correlation coefficients (Pearson’s r) between forest cover and all forest-dwelling mammal species richness **c**, all amphibian species richness **d**, and all reptile species richness **e**.

Regional correlation results over the seven forested biomes were distinguishable (Fig. 3), as reflected by geographic variation from the poles to the equator. Specifically, for the three taxonomic groups, biomes such as Taiga (also called boreal forests, BF), temperate conifer forests (TCF), and tropical and subtropical coniferous forests (TSCF) showed a downward trend more in line with the global overall trend (Fig. 3c–e). The dynamics of vertebrate-forest correlation patterns over time in temperate broadleaf and mixed forest (TBMF) and Mediterranean forests, woodlands and scrub (MFWS) were complex (Fig. 3c–e). The remaining two biomes, tropical and subtropical dry broadleaf forests (TSDBF) and tropical and subtropical moist broadleaf forests (TSMBF), instead showed a gradual climbing trend in their correlation curves (Fig. 3c–e). Meanwhile, in all the seven forested biomes, the overall forest cover of the areas impacted by PAs was higher than that of areas that did not receive protection (except BF), a pattern that has persisted since the initial establishment of PAs at a global level (Fig. 3b).

### Impact of protected areas on vertebrate extinction debts

**Fig. 4:**
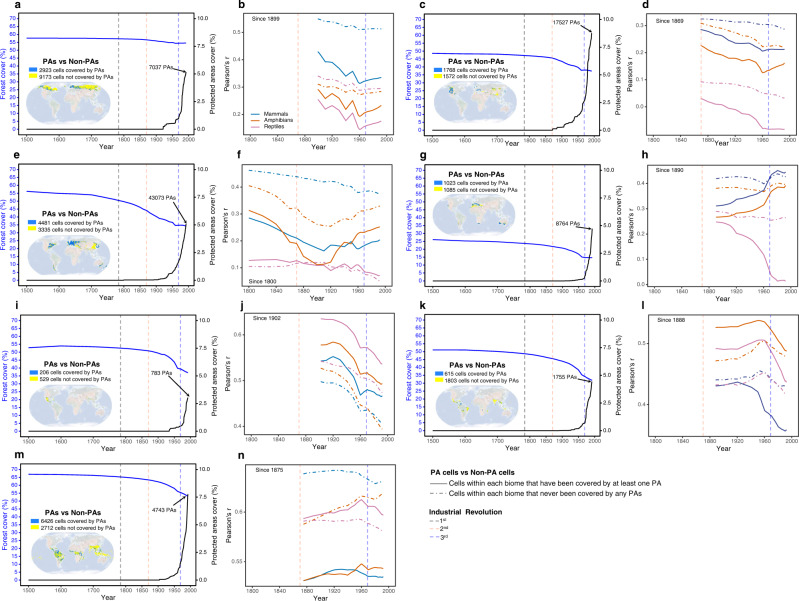
Temporal dynamics of regional protected areas, forest cover and correlation coefficients. The yearly percentage area change in forest cover and the change in the number of protected areas in seven forested biomes (**a**, **c**, **e**, **g**, **i**, **k**, **m**). Comparisons of the changes in correlation coefficients (Pearson’s r) between the cells within each biome that were covered by at least one protected area and the cells that had never been covered by any protected area **b**, **d**, **f**, **h**, **j**, **l**, **n**. The seven forested biomes correspond to boreal forests/Taiga **a**, **b**, temperate conifer forests **c**, **d**, temperate broadleaf & mixed forests **e**, **f**, Mediterranean forests, woodlands & scrub **g**, **h**, tropical & subtropical coniferous forests **i**, **j**, tropical & subtropical dry broadleaf forests **k**, **l** and tropical & subtropical moist broadleaf forests **m**, **n**.

Furthermore, in all seven forested biomes, PAs have positive and timely effects on maintaining or increasing forest cover (Fig. 4a, c, e, g, i, k, m). For mammals, PAs established before 1992 had positive effects only in MFWS (Fig. 4h) and TSCF (Fig. 4j). For amphibians, PAs had positive effects in TSCF (Fig. 4j) and TSDBF (Fig. 4l); for reptiles, PAs contributed positively to TBMF (Fig. 4f), TSCF (Fig. 4j), TSDBF (Fig. 4l) and TSMBF (Fig. 4n). In the other cases, however, the positive role of protected areas was not evident (Fig. 4).

## Discussion

Deforestation is one of the main causes of extinction for many forest-dwelling vertebrates^24^; it means habitat destruction, increased risk of predation and hunting, reduced food supply, etc. The present study proved that extinction debts were triggered around the mid-19^th^ century and have accumulated over the past half-millennium for the three forest-dwelling vertebrate groups (Fig. 2). The existence of debt evidence implied that the negative effects of deforestation on the diversity and distribution of global terrestrial vertebrates might require dozens or hundreds of years to realize or relax (i.e., biotic relaxation).

The low correlations between global reptile richness and forest area size implied that reptile species were not as sensitive to the loss of forest cover (Figs. 2 and 3e). This is particularly true due to the fact that many reptile species can inhabit dry and desert regions where forest cover is very low^25,26^. As an example, the world’s most diverse lizard fauna are found in the desert areas of western Australia^27^. In contrast, amphibians (especially for threatened categories) typically require diverse habitat types with a mixture of grasslands, forests and water bodies^28–30^, while many mammals (especially those large-bodied ones) occupy large forested areas as home ranges for reproduction, survival and food^31–33^, thus becoming sensitive to changes in both forest and climate.

In seven forested biomes, we found stronger signals of extinction debt for conifer-dominated biomes, particularly in boreal and temperate conifer forests, which are home to many threatened mammals, such as the Siberian tiger, one of the most endangered animals in the world^24^. These coniferous forests are also areas that will be more vulnerable to global warming in the future^34^. In contrast, in tropical and subtropical broadleaf forests (both moist and dry types), no significant extinction debt signals were detected in amphibian, reptile and mammalian taxa, presumably for the following reasons: i) compared to other biomes, tropical and subtropical broadleaf forests have higher habitat connectivity and fewer past disturbances (Fig. 3b); ii) the two biomes have many new species that have only been discovered in recent decades, and these recently discovered species are characterized by small body sizes (e.g., amphibians and reptiles), low dispersal ability, narrow ecological niches, and high endemicity; therefore, the overall decline in forest cover in both biomes does not have a strong impact on these narrowly distributed species^35^; iii) biomes with the highest species richness have gradually adapted to present-day landscape structures and thus may be more in line with current habitat than historically^31^. However, we suspect that this phenomenon is not permanent and may be transient. On the one hand, the forest-vertebrates correlation in the two biomes has decreased since the 20th century (Fig. 3), and on the other hand, extreme weather events have been occurring in recent years, such as fires in the Amazon, disturbing the hard-won equilibrium^36^.

Our study showed that the start of modern industrialization in the mid-19th century was the triggering point of extinction debts, as the correlations declined after that point in time (Fig. 2). This is also evidenced by a well-fitted linear relationship shown by the accelerating trend of the Kyoto Human-caused greenhouse gas emissions, which represents industrialization levels, and the accelerating decline of biodiversity-habitat relationships (Fig. 2c). Moreover, at exactly the same moment, global vertebrates were predicted to undertake rapid population decline, as reported in a recent population-level study^37^. Such a striking timing coincidence revealed that human industrialization in the nineteenth century triggered and accumulated delayed extinction of global vertebrates, as evidenced in our present study, and one direct piece of evidence of the debts is the rapid population decline of species, as found in a previous study^37^.

The gradational decline of correlations for the richness of global forest-dwelling vertebrate species regarding the size of forested habitat from the past to the present implied that species diversity and the surrounding environment are subject to a process of disequilibrium, resulting in the time-lagged responses of biodiversity in reaction to forest change. This was confirmed by the comparative analysis of PA cells and non-PA cells. The impact of global PAs on the mitigating accumulated vertebrate extinction debt is not as immediate as that of mitigating reduced forest cover but rather suffers from pronounced time-lag effects (Fig. 4). Generally, governments tend to approve PAs where extinction events have already occurred^38^, so the timing of protected area establishment and debt emergence itself was mismatched. However, from 1950 onwards, the correlation coefficients in PAs of the majority of the biomes were raised again, suggesting that the downward trend in debt has eased and that it may be dozens to hundreds of years before PAs can become functionally effective. This striking finding suggested that conservation strategies should be refocused from PAs towards areas experiencing rapid losses of forested habitat (e.g., Figs. 1, 3, 4).

There are limitations that must be mentioned. We borrowed the idea of ‘stable habitat’ in our analysis of the effectiveness of protected areas to compare the differences in extinction debt signals between protected and nonprotected areas. However, the ‘past habitat’ approach used in this study for detecting extinction debts is weaker than the ‘past communities’ method, which utilizes past species richness information^8^. Furthermore, although we sought a compromise for the differences between the three algorithms (forward, backward and JP), there were biases and uncertainties in some regions of the reconstructed forest dataset (see Supplementary Fig. 1). Data uncertainty, however, did not alter our important conclusions, as indicated by the consistency of correlation results generated from the three different algorithms at global scales (Supplementary Figs. 1–3). Furthermore, we must note that there are inevitable issues of spatial and temporal autocorrelation in analyzing annual trends of forest cover at the global scale. Since we have attempted to model the influence of temporal patterns, we have considered dealing with spatial rather than temporal autocorrelation; the latter is more valuable in examining the signals of declining forest resilience under climate change^39^.

Given that the correlation values are declining from the past to the present and because of the synergistic effects of future climate warming and deforestation, it is speculated that the correlation between species richness and forest area size would further decline in the future periods for these terrestrial taxonomic groups. From the current time to the future, because of the double effects of climatic and habitat changes mentioned above, time-delayed species losses will be accelerating. As a result, the estimation of species extinction is more prone to underestimation. From a methodological perspective, adequate dynamic statistical models^12,40^ should be developed to better capture the transient nature of the species extinction process to tackle the complex trajectories of biodiversity loss in a changing world.

Last and more importantly, to effectively mitigate the negative effects of historical forest loss on species extinction over the past 500 years, our study underscores the importance of afforestation for increasing the amount and improving the connectivity of forested habitat and accordingly contributing to the effect of “immigration credits”^7,41^. Other than biodiversity conservation, the recovery of forest habitat would be of great value to maintain ecosystem services and stability^21,42^. The future accrual of forested areas, in part through afforestation efforts, is anticipated to substantially offset the dual threats of forest loss and climate warming to the survival of global land vertebrate taxa.

## Methods

### Global distributions of forest-dwelling vertebrate species

The spatial distributional data in Esri shapefile format for global terrestrial vertebrate groups, including mammals, amphibians, and reptiles, were compiled from the International Union for Conservation of Nature (IUCN) Red List website^24^. For subsequent analyses, we selected terrestrial forest-dwelling species in each taxonomic group by excluding species only inhabiting marine, freshwater or other non-forest habitats based on the IUCN habitat information. This means that species with forest as their primary habitat and other habitat types, such as shrubs and grasslands, as secondary habitats are defined as forest-dwelling species^17^. We further selected only parts of each species’ range designated as extant, either native or reintroduced, and either seasonally resident or breeding or non-breeding^43^. Finally, a total of 4278 mammal, 6047 amphibian, and 6120 reptile species were selected for analysis. We then overlapped the distributional range of each species over a grid system with a spatial resolution of 0.5°×0.5° geographic degrees that covered the world land surface (excluding Antarctica) to record its presence/absence information in each grid cell. The cumulative presence-absence information for all the species (including data-deficit species—DD), as well as for critically endangered (CR), endangered (EN), vulnerable (VU), near threatened (NT), and least concern (LC) species, are used for generating species richness patterns and subsequent analyses by overlaying their range maps (Supplementary Figs. 4–6). Extinct (EX) and wild-extinct (EW) forest-dwelling species were not included in the analysis because they failed to survive to the present day.

### Global historical forest cover

Historical global forest cover data dating back to the year 1500 with a spatial resolution of 0.5° × 0.5° were reconstructed from the Land Use Harmonization (LUH; version 3.1) database (http://luh.umd.edu/data.php)^44^ and the current-time European Space Agency Climate Change Initiative (CCI) land-cover map (http://www.esa-landcover-cci.org/), with two earth system models (hereafter called algorithms), i.e., backward (1500–2005) and forward (1500–2005)^45^. For example, for the backward algorithm, starting from the CCI land-cover map, we estimated annual changes in different land use types between two consecutive years based on the LUH data by a backward “proportional allocation rule”^46^. That is, the annual historical changes in urban, cropland and pasture areas in each grid cell were calculated as percentages in a backward and recursive way back to 1500 by using the ratios of existing natural grassland to forest for different land use types^47^. The historical gain or loss of forest cover thus was proportionally related to the overall changes in urban, cropland and pasture areas^46^. To implement the forward method, the initial map for 1500 was obtained from the historical vegetation functional map reconstructed by Pongratz et al.^48^ for the years 800–1992 (hereafter, this dataset is referred to as JP). More details about the implementation of the backward and forward methods are well described in ref. ^45^.

Based on the above three datasets, we performed subsequent separate global and regional correlation analyses. At the same time, to reduce the uncertainties caused by the reconstructing algorithm, we adopted an ensemble approach, i.e., calculated the mean value in each 0.5° grid cell as the present output. Simultaneously, because the time periods of the three datasets are not identical^45^, we selected only the overlapping time periods, i.e., 1500–1992, at one-year intervals and mapped 493 consecutive global forest cover layers using an ensemble approach.

### Statistics and reproducibility

To test whether there were extinction debts for forest-dwelling vertebrates over the past 500 years, we utilized a modified t-test, which can take spatial autocorrelation into account to assess the correlation between species richness and forest cover. The correlation values (Pearson’s r) were used to determine whether historical forest change would result in the disequilibrium of diversity-habitat associations. If the correlation was found to decrease, the evidence of diversity-habitat disequilibrium was confirmed, and the evidence of extinction debts was justified. To further explore the extent to which the three industrial revolutions have affected the extinction debt of forest vertebrates through forest cover change, we calculated the decade change of correlation coefficients between all mammal, amphibian and reptile richness and forest cover.

Generally, the timelines of the three industrial revolutions are 1500 to 1784 (start of the first industrial revolution through the introduction of the first mechanical loom), 1784 to 1870 (start of the second industrial revolution through the introduction of the first assembly line) and 1870 to 1969 (start of the third industrial revolution through the use of electronic and IT systems)^49^. Linear models were fitted using Kyoto human-caused greenhouse gas (GHG) yearly emissions (1850–2017) statistics by the Potsdam Institute for Climate Impact Research (PIK)^50^ and the calculated correlation coefficients. PIK is an ideal and independent validation dataset for our study because it did not include emissions from land use, land use change and forestry (LULUCF), all of which are sources and sinks of emissions. The above analyses for backward, forward and JP datasets can also be found in Supplementary Figs. 1–3.

To explore the role that PAs actually play in the balanced relationship between forest cover and vertebrate richness, we focused our study scale on biomes^51^. Globally, there are 867 terrestrial ecoregions classified into 14 different biomes. Among them, seven biomes are forested, that is, boreal forests/Taiga (BF), temperate conifer forests (TCF), temperate broadleaf & mixed forests (TBMF), Mediterranean forests, woodlands & scrub (MFWS), tropical & subtropical coniferous forests (TSCF), tropical & subtropical dry broadleaf forests (TSDBF), and tropical & subtropical moist broadleaf forests (TSMBF).

We downloaded the shapefiles of 257,348 global protected areas from the World Database on Protected Areas^52^. Based on the recorded status year, we extracted 57,113 terrestrial PAs proposed, established or designated before 1992^53^ (Supplementary Fig. 7). Furthermore, we intersected each of the forested biomes with a 0.5-degree grid system. We then intersected the grid cells within the range of each biome with all 57,113 protected areas and divided them into those that had ever received conservation services and those that had never received them (Supplementary Fig. 8). In this process, there were cases where some PAs were included in the calculation by more than one biome. Since the biome analysis is independently performed, there is no concern about the existence of pseudo-replication.

Considering that 0.5-degree georeferenced grids exist in spatially unequal area projections, the forest cover of each biome was counted based on a weighted sum of the area and percent cover of each grid cell. In addition, we calculated the changes in the percentage and number of PAs in each biome from 1800 to 1992 (the start time depends on the status year of PAs within each biome). The correlation coefficients for each vertebrate taxon within each biome in general, grid cells within each biome that received protection and grid cells that did not receive protection were calculated separately.

All analyses were performed in R v.4.0.2^54^ and ArcGIS v.10.8 (http://www.esri.com/).

### Reporting summary

Further information on research design is available in the Nature Portfolio Reporting Summary linked to this article.

## Supplementary information

Supplementary Information

Reporting Summary

## Data Availability

The expert-derived range maps of mammals, amphibians and reptiles are freely accessible from the IUCN Red List Portal (https://www.iucnredlist.org/resources/spatial-data-download). The global reconstructed yearly forest cover from 1500 to 1992 at a resolution of 0.5 degrees in TIFF format based on backward and forward algorithms can be requested from ref. ^45^, while the JP reconstructed forest cover within the same time periods can be requested from ref. ^48^. Global protected areas can be freely accessed from the World Database on Protected Areas (https://www.protectedplanet.net/en/thematic-areas/wdpa). Biome polygons are available at the RESOLVE Ecoregions database (https://ecoregions.appspot.com/). Kyoto human-caused greenhouse gas (GHG) emissions (1850–2017) statistics by the Potsdam Institute for Climate Impact Research (PIK) can be freely accessed from the Climate Watch platform (https://www.climatewatchdata.org/ghg-emissions). The ensemble weighted forest cover dataset as well as the species richness maps of mammals, amphibians and reptiles grouped by IUCN Red List categories constructed in the present study are publicly available from the GitHub repository (https://github.com/optiforziyan/vertebrates-extinction-debt).

## Code availability

The codes for visualizing Figs. 1–4 of the main text are available from the GitHub repository (https://github.com/optiforziyan/vertebrates-extinction-debt).
