# Supplementary material for: Half-millennium evidence suggests that extinction debts of global vertebrates started in the Second Industrial Revolution
Source: Commun Biol. 2022 Dec 13;5:1311. doi: 10.1038/s42003-022-04277-w (PMC9747783; doi:10.1038/s42003-022-04277-w)
Supplement: Supplementary file 1 — Supplementary Information [file 42003_2022_4277_MOESM1_ESM.pdf]

Half-millennium evidence suggests that extinction debts of global vertebrates started in the Second Industrial Revolution

Ziyan Liao[1], Shushi Peng[2], Youhua Chen[1]*

[1]China-Croatia “Belt and Road” Joint Laboratory on Biodiversity and Ecosystem Services, Chengdu Institute of Biology, Chinese Academy of Sciences, Chengdu 610041, China
[2]Sino-French Institute for Earth System Science, College of Urban and Environmental Sciences, and Laboratory for Earth Surface Processes, Peking University, Beijing 100871, China

*Corresponding author: Youhua Chen (chenyh@cib.ac.cn)

Supplementary Information
This file includes Supplementary Figures 1-7

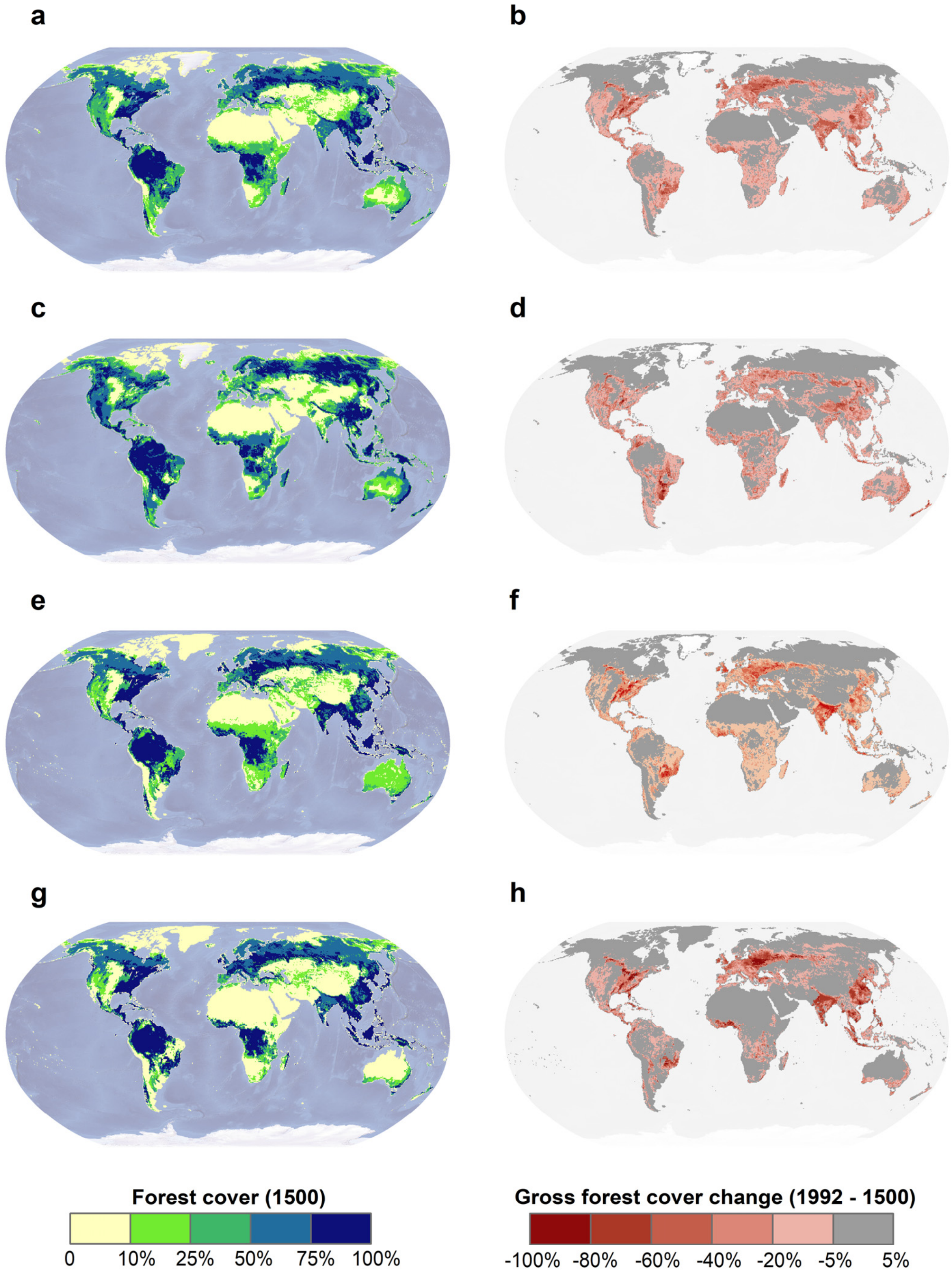

Supplementary *Fig. 1. Overview of global forest cover change based on four reconstructed methods. Forest cover in the year 1500 based on an ensemble approach (a), backward (c), forward (e) and JP (g) algorithms. Percent gross forest cover change (unit: %) from 1500 to 1992 based on an ensemble approach (b), backward (d), forward (f), and JP (h) algorithms. The projection system is the Robinson projection and the base map was obtained from Natural Earth (http://www.naturalearthdata.com/).*

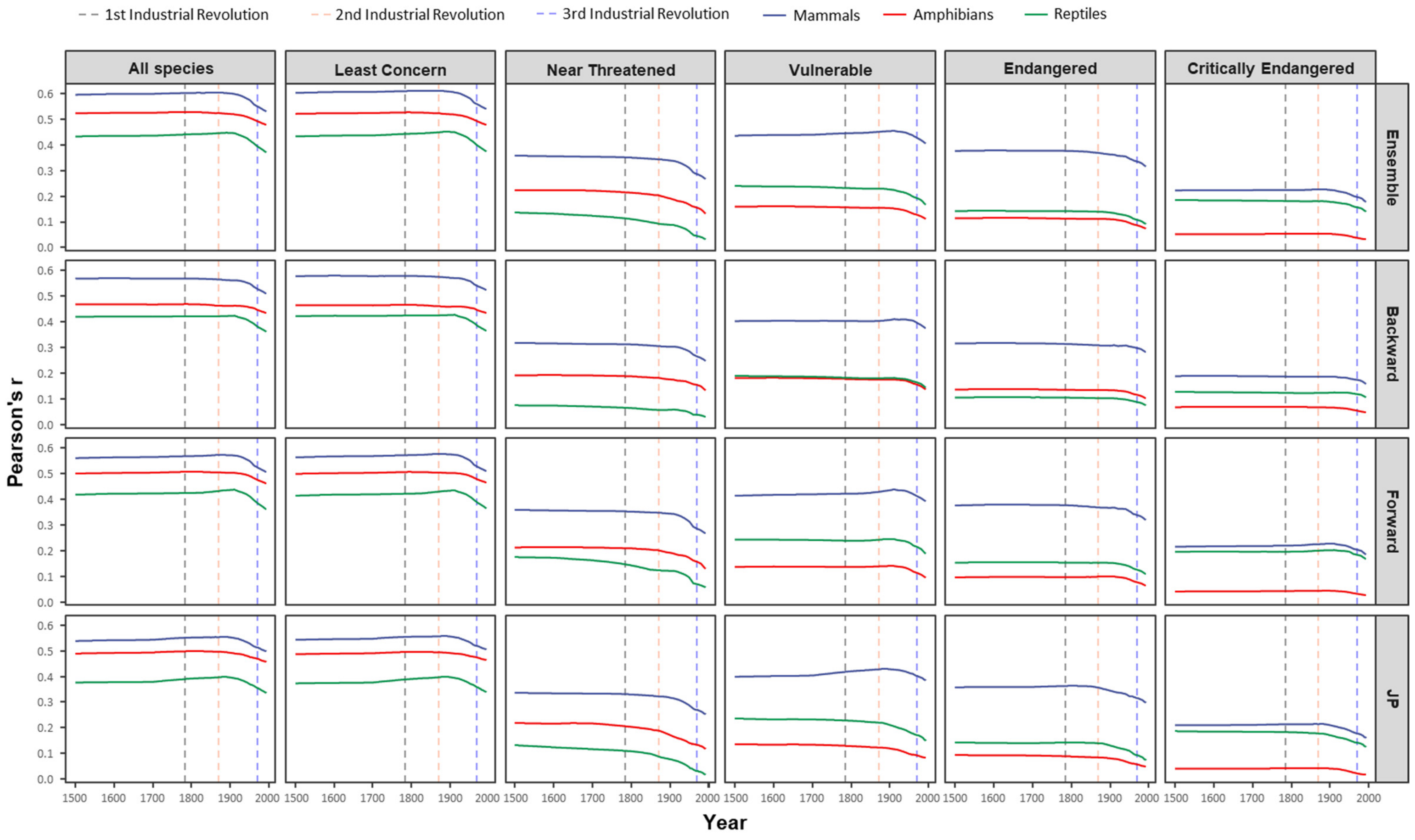

Supplementary *Fig. 2. Correlation coefficients (Pearson's r) between the richness of contemporary forest-dwelling vertebrate taxa and the area size of historical forested habitat at the global scale from 1500 to 1992 (unit: per year).*

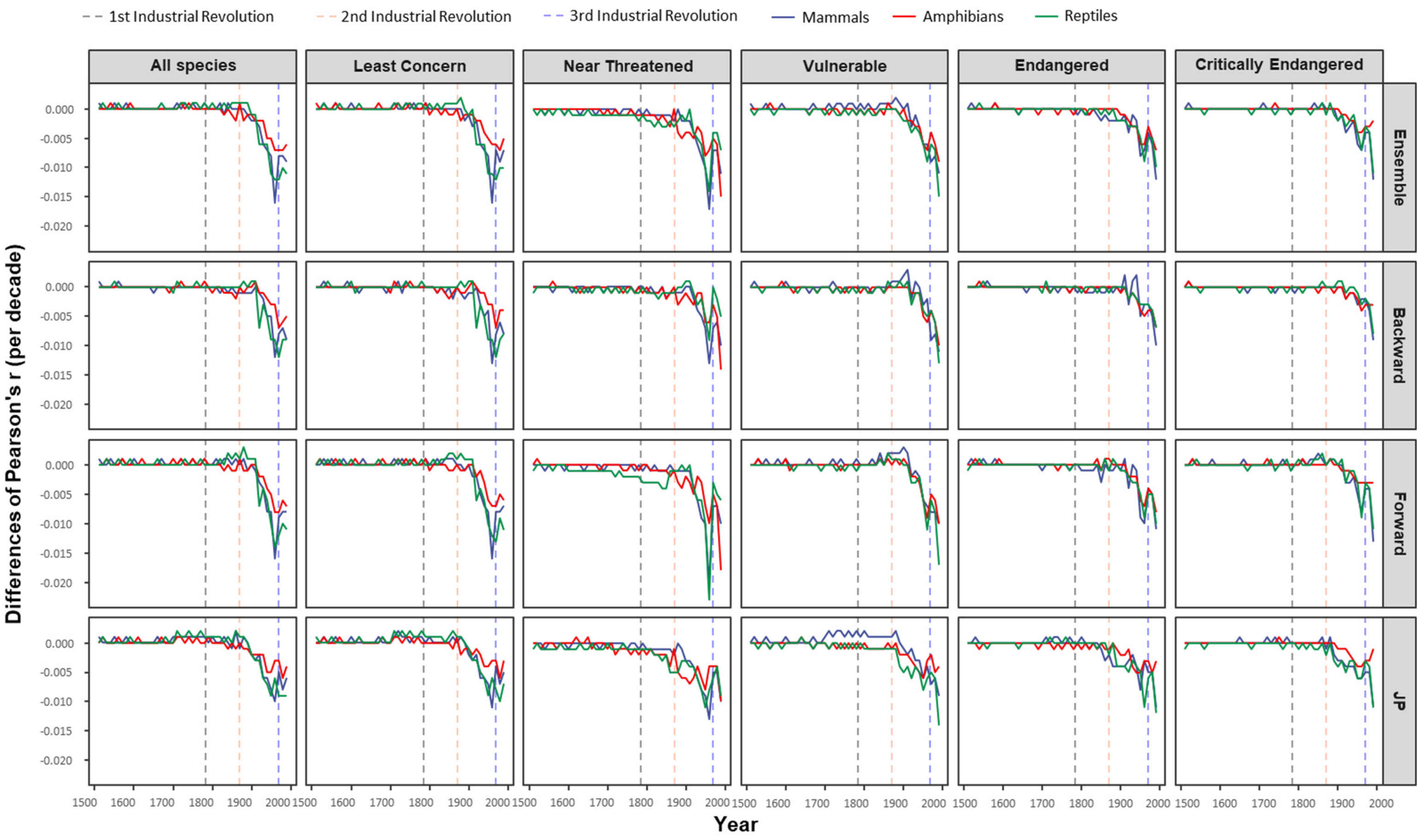

Supplementary *Fig. 3. Difference in correlation coefficients (Pearson's r) between the richness of contemporary forest-dwelling vertebrate taxa and the area size of historical forested habitat at the global scale from 1500 to 1992 (unit: per decades).*

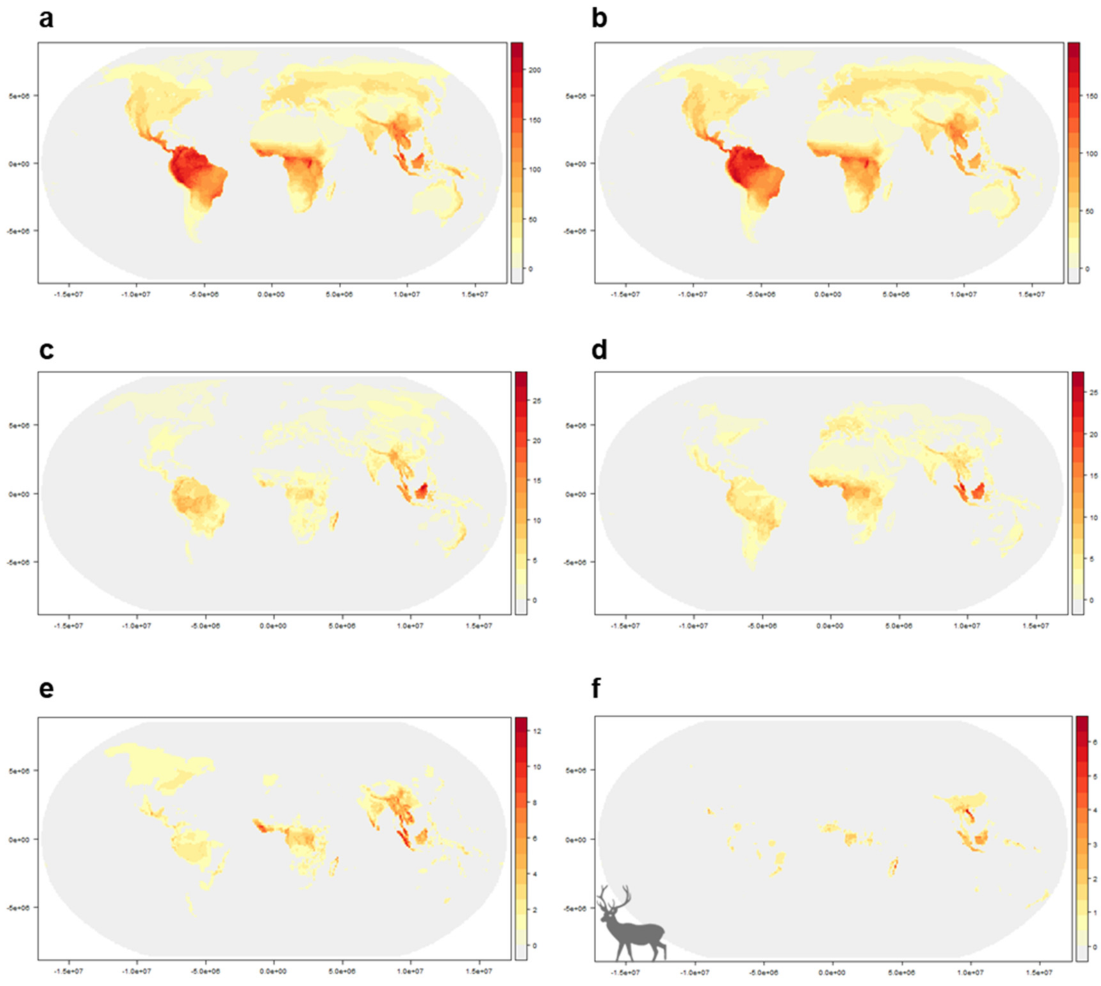

Supplementary *Fig. 4. Species richness map of different IUCN Red List categories for global forest-dwelling mammals. All species richness including data-deficit species (a). Least concern species richness (b). Vulnerable species richness (c). Near Threatened species richness (d). Endangered species richness (e). Critically Endangered species richness (f). These maps were created using levelplot function of 'rasterVis' R package.*

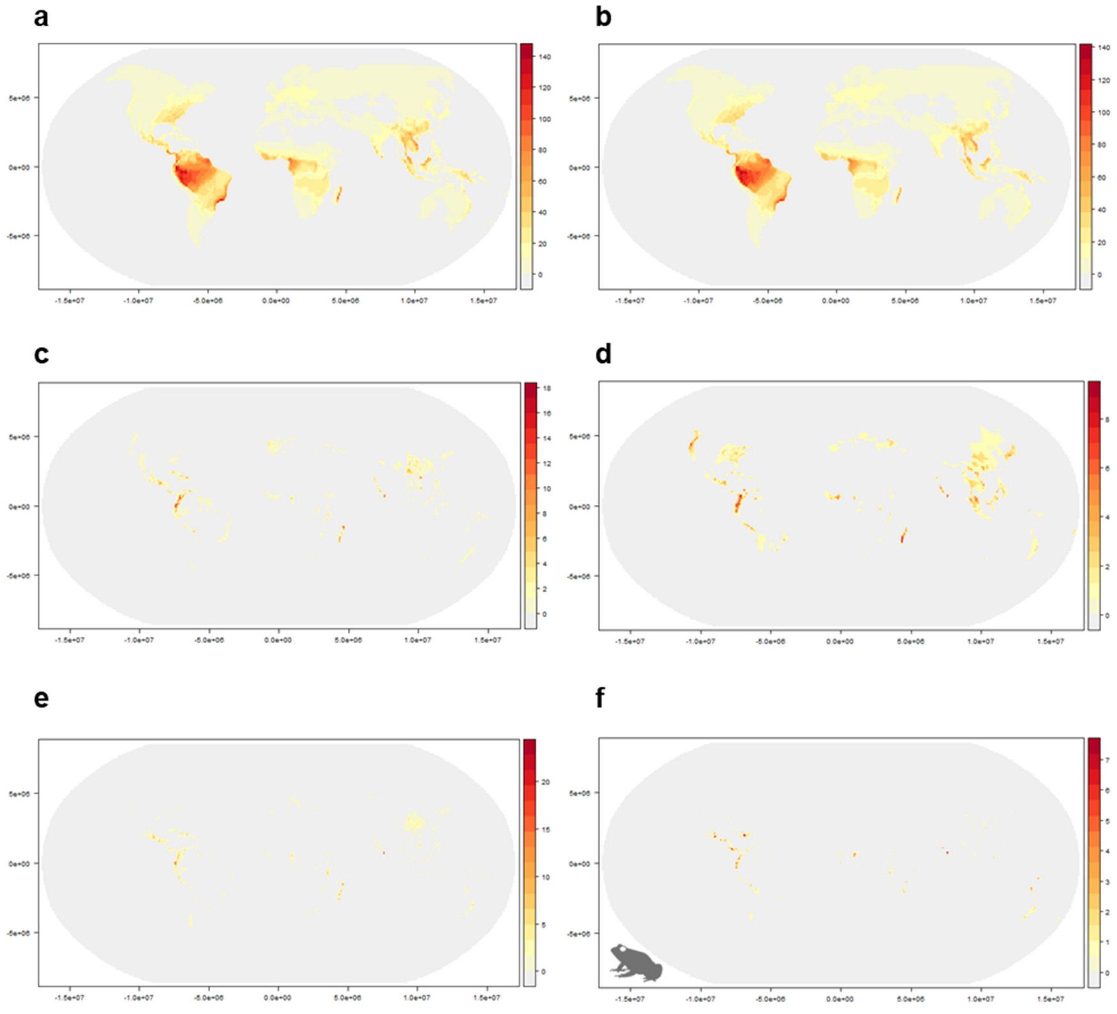

Supplementary *Fig. 5. Species richness map of different IUCN Red List categories for global forest-dwelling amphibians. All species richness including data-deficit species (a). Least concern species richness (b). Vulnerable species richness (c). Near Threatened species richness (d). Endangered species richness (e). Critically Endangered species richness (f). These maps were created using levelplot function of 'rasterVis' R package.*

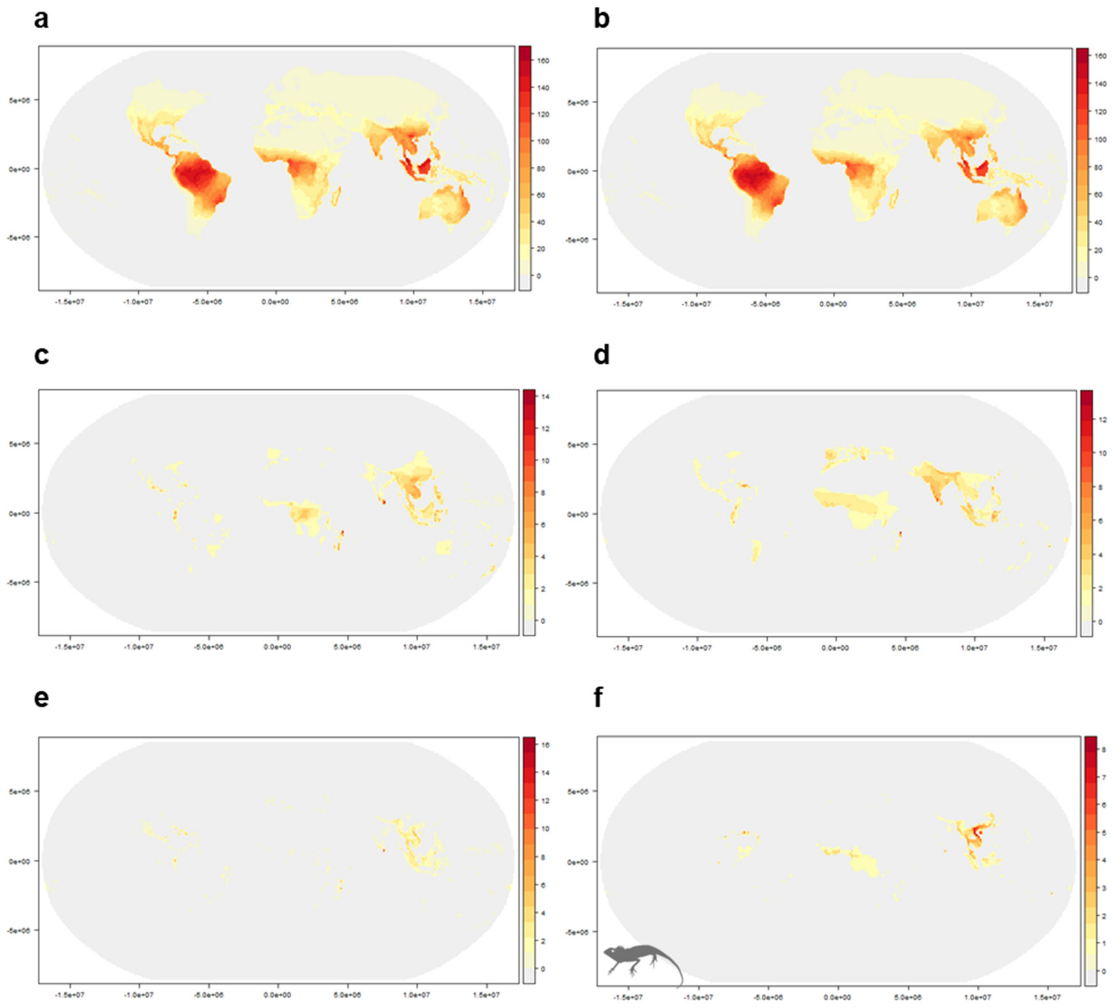

Supplementary *Fig. 6. Species richness map of different IUCN Red List categories for global forest-dwelling reptiles. All species richness including data-deficit species (a). Least concern species richness (b). Vulnerable species richness (c). Near Threatened species richness (d). Endangered species richness (e). Critically Endangered species richness (f). These maps were created using levelplot function of 'rasterVis' R package.*

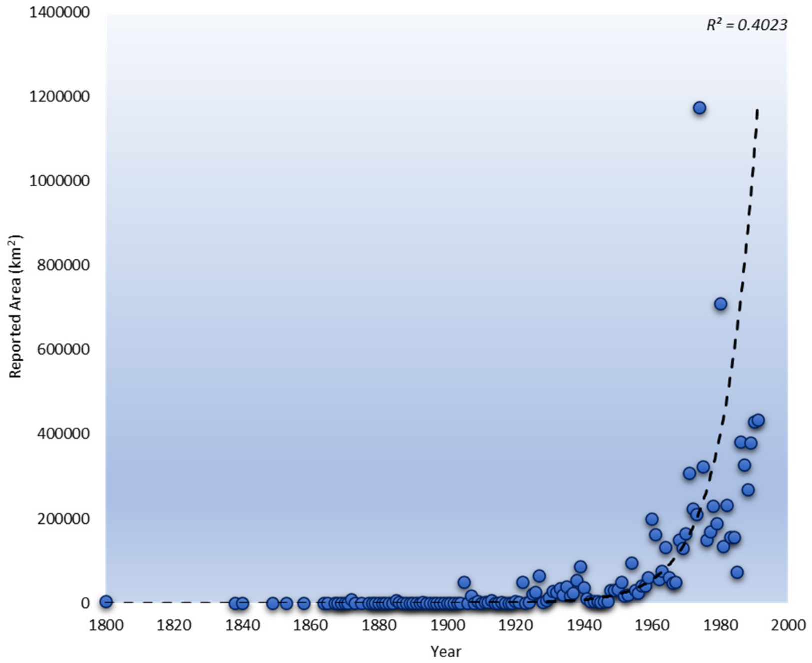

Supplementary *Fig. 7. Total area of proposed, established and designated protected areas from 1800 to 1992. An exponential regression is used to characterize the overall trend.*

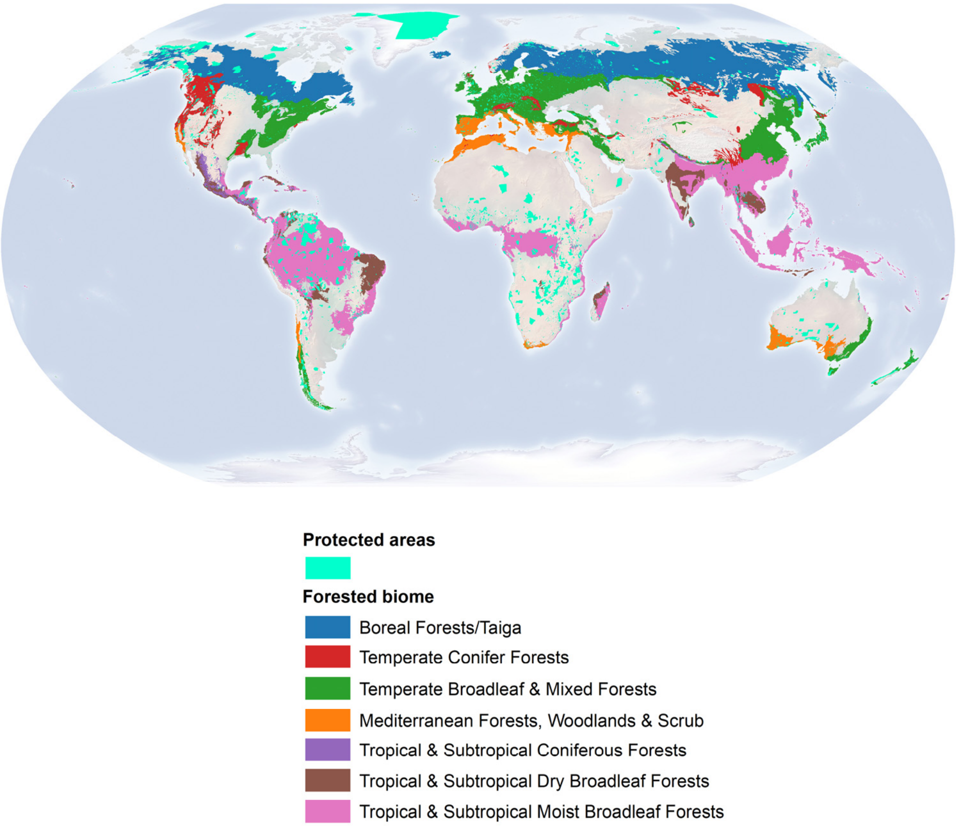

***Supplementary** Fig. 8. Overview of the distribution patterns of 57,113 terrestrial protected areas established before the year 1992.*
