# Supplementary material for: Half-millennium evidence suggests that extinction debts of global vertebrates started in the Second Industrial Revolution
Source: Commun Biol. 2022 Dec 13;5:1311. doi: 10.1038/s42003-022-04277-w (PMC9747783; doi:10.1038/s42003-022-04277-w)
Supplement: Supplementary file 2 — Reporting Summary [file 42003_2022_4277_MOESM2_ESM.pdf]

nature portfolio

Corresponding author(s): Youhua Chen

Last updated by author(s): Nov 8, 2022

Reporting Summary

Nature Portfolio wishes to improve the reproducibility of the work that we publish. This form provides structure for consistency and transparency in reporting. For further information on Nature Portfolio policies, see our Editorial Policies and the Editorial Policy Checklist.

Statistics

For all statistical analyses, confirm that the following items are present in the figure legend, table legend, main text, or Methods section.

n/a	Confirmed	
☐	☒	The exact sample size (*n*) for each experimental group/condition, given as a discrete number and unit of measurement
☒	☐	A statement on whether measurements were taken from distinct samples or whether the same sample was measured repeatedly
☒	☐	The statistical test(s) used AND whether they are one- or two-sided<br>*Only common tests should be described solely by name; describe more complex techniques in the Methods section.*
☐	☒	A description of all covariates tested
☒	☐	A description of any assumptions or corrections, such as tests of normality and adjustment for multiple comparisons
☐	☒	A full description of the statistical parameters including central tendency (e.g. means) or other basic estimates (e.g. regression coefficient) AND variation (e.g. standard deviation) or associated estimates of uncertainty (e.g. confidence intervals)
☒	☐	For null hypothesis testing, the test statistic (e.g. *F*, *t*, *r*) with confidence intervals, effect sizes, degrees of freedom and *P* value noted<br>*Give P values as exact values whenever suitable.*
☒	☐	For Bayesian analysis, information on the choice of priors and Markov chain Monte Carlo settings
☒	☐	For hierarchical and complex designs, identification of the appropriate level for tests and full reporting of outcomes
☐	☒	Estimates of effect sizes (e.g. Cohen's *d*, Pearson's *r*), indicating how they were calculated

Our web collection on statistics for biologists contains articles on many of the points above.

Software and code

Policy information about availability of computer code

Data collection	The expert-derived range maps of mammals, amphibians and reptiles are freely accessible from the IUCN Red List Portal (https://www.iucnredlist.org/resources/spatial-data-download). The global reconstructed yearly forest cover from 1500 CE to 1992 CE at a resolution of 0.5 degrees in TIFF format based on backward and forward algorithms can be requested from Peng et al., (2017), while the JP reconstructed forest cover within the same time periods can be requested from Pongratz et al., (2008). Global protected areas can be freely accessed from the World Database on Protected Areas (https://www.protectedplanet.net/en/thematic-areas/wdpa). Biome polygons are available at the RESOLVE Ecoregions database (https://ecoregions.appspot.com/). Kyoto human-caused greenhouse gas (GHG) emissions (1850-2017) statistics by the Potsdam Institute for Climate Impact Research (PIK) can be freely accessed from the Climate Watch platform (https://www.climatewatchdata.org/ghg-emissions).
Data analysis	All analyses were performed in R v.4.0.2 and ArcGIS v.10.8. In R, the packages 'sf', 'raster', ''ggplot2', 'ggsci', 'ggpmisc' and 'doParallel' were mainly used.

For manuscripts utilizing custom algorithms or software that are central to the research but not yet described in published literature, software must be made available to editors and reviewers. We strongly encourage code deposition in a community repository (e.g. GitHub). See the Nature Portfolio guidelines for submitting code & software for further information.

Data

Policy information about availability of data

All manuscripts must include a data availability statement. This statement should provide the following information, where applicable:

- Accession codes, unique identifiers, or web links for publicly available datasets
- A description of any restrictions on data availability
- For clinical datasets or third party data, please ensure that the statement adheres to our policy

The ensemble weighted forest cover dataset as well as the species richness maps of mammals, amphibians and reptiles grouped by IUCN Red List categories constructed in the present study are publicly available from the GitHub repository (https://github.com/optiforziyan/vertebrates-extinction-debt). The codes for visualizing Figures 1~4 of the main text are available from the GitHub repository (https://github.com/optiforziyan/vertebrates-extinction-debt).

Human research participants

Policy information about studies involving human research participants and Sex and Gender in Research.

Reporting on sex and gender	Not applicable
Population characteristics	Not applicable
Recruitment	Not applicable
Ethics oversight	Not applicable

Note that full information on the approval of the study protocol must also be provided in the manuscript.

Field-specific reporting

Please select the one below that is the best fit for your research. If you are not sure, read the appropriate sections before making your selection.

☐ Life sciences ☐ Behavioural & social sciences ☒ Ecological, evolutionary & environmental sciences

For a reference copy of the document with all sections, see nature.com/documents/nr-reporting-summary-flat.pdf

Ecological, evolutionary & environmental sciences study design

All studies must disclose on these points even when the disclosure is negative.

Study description	This study shows that the beginning of the Second Industrial Revolution (the mid-19th century) was the earliest signal of cumulative extinction debts for global forest-dwelling vertebrate groups.
Research sample	The datasets used includes the geographical distributions of 6120 reptiles, 4278 mammals and 6047 amphibians, annual forest cover data from 1500 to 1992, 57,113 protected areas and 14 biomes.
Sampling strategy	No data was specifically sampled for this study.
Data collection	All data are collected from free on-line data platforms.
Timing and spatial scale	The timing scale of the analysis was from 1500 to 1992, and the spatial scale was global.
Data exclusions	We selected only terrestrial forest-dwelling species in each taxonomic group, by excluding species inhabiting marine, freshwater or other non-forest habitats based on the IUCN habitat information.
Reproducibility	No experiments were used. All the results of this study can be reproducible by R codes.
Randomization	Local correlation patterns are based on the WWF forested biomes which might lead to partial marginal effects.
Blinding	n/a

Did the study involve field work? ☐ Yes ☒ No

Reporting for specific materials, systems and methods

We require information from authors about some types of materials, experimental systems and methods used in many studies. Here, indicate whether each material, system or method listed is relevant to your study. If you are not sure if a list item applies to your research, read the appropriate section before selecting a response.

Materials & experimental systems

n/a	Involved in the study
☒	☐ Antibodies
☒	☐ Eukaryotic cell lines
☒	☐ Palaeontology and archaeology
☒	☐ Animals and other organisms
☒	☐ Clinical data
☒	☐ Dual use research of concern

Methods

n/a	Involved in the study
☒	☐ ChIP-seq
☒	☐ Flow cytometry
☒	☐ MRI-based neuroimaging
